# Effect of age and diet on carcass and meat quality in ewes

**DOI:** 10.1016/j.heliyon.2023.e22350

**Published:** 2023-11-15

**Authors:** Cecilia Rivera-Bautista, Alicia Grajales-Lagunes, Alejandro Relling, Alfonso Chay-Canul, Anayeli Vazquez-Valladolid, Einar Vargas-Bello-Pérez, Héctor A. Lee-Rangel

**Affiliations:** a Facultad de Agronomía y Veterinaria - Centro de Biociencias Universidad Autónoma de San Luis Potosí, Soledad de Graciano Sánchez, San Luis Potosí, 78321, Mexico; b Facultad de Ciencias Químicas, Universidad Autónoma de San Luis Potosí, San Luis Potosí, 78000, Mexico; c Department of Animal Science, The Ohio State University, Ohio Agricultural Research and Development Center (OARDC), Wooster, OH, 44691, USA; d División Académica de Ciencias Agropecuarias, Universidad Juárez Autónoma de Tabasco, Villahermosa, 86280, Mexico; e Department of Animal Sciences, School of Agriculture, Policy and Development, University of Reading, P.O. Box 237, Earley Gate, Reading, RG6 6EU, UK

**Keywords:** Cull ewes, Diet, Wool ewes, Meat quality, Yearling, Carcass, Meat color, Tenderness

## Abstract

This study elucidated the effect of age and diet on carcass characteristics and meat quality parameters of Rambouillet ewes. Forty ewes (n = 20 yearling ewes and n = 20 cull ewes) were fed with alfalfa hay (AH) or a 100 % concentrate diet (CD). Treatments were: a) 10 cull ewes were fed only with AH, b) 10 yearling ewes were fed only with AH, c) 10 cull ewes were fed with CD, d) 10 yearling ewes were fed with CD. Productive performance, carcass and meat quality were analyzed. Animals had ten days for adaptation and 35 days were used to collect data. Dry matter intake was greater (P < 0.05) for CD. Feed conversion rates were not affected by treatments. The pH at 45 min and 24 h, carcass length, leg length, leg width, thorax width, and thorax perimeter were not affected by treatments. Hot carcass weight was heavier (P < 0.05) in cull ewes, cold carcass weight was increased (P < 0.05) with CD. Carcass yield (CY) was heavier in CD (P < 0.05). Cull ewes had greater (P < 0.05) lean CIELAB L*, a*, b*, c*, and h* values compared to yearling ewes. The color changes increased with age at five days (P < 0.05), but a decrease (P < 0.05) with diet was observed at ten days. Cathepsins B, B + L, and Lowry protein content were not affected by treatments. In conclusion, feeding cull ewes with concentrate diets may enhance body weight gain and carcass yield compared to a diet based on 100 % alfalfa hay.

## 1 Introduction

Sheep meat consumption and market in Mexico has been dominated by a traditional dish known as *Barbacoa* [1]. Moreover, about 40 % of slaughtered animals are adults or cull animals with low body condition score and low live weight [2,3]. The sale of adult or cull animals represents an extra income for farmers [[3], [4], [5]]. Moreover, the selling price could be better valued if carcass traits are improved [4,5]. However, factors affecting carcass characteristics and carcass value of this type of animal are rarely studied [4,5].

Ewes are culled around 5–6 years of age and they are characterized by poor body condition [6]. The meat of culled ewes is tough, fibrous in texture and high in insoluble collagen content [7] resulting in low consumer preference. Short-term re-feeding of culled ewes before slaughtering results in significant weight gains, and this could result in improved carcass yields [8]. Also, it has been reported that decreasing meat shear force and quantity of soluble collagen increases consumer acceptability [9,10].

With age, ewe's carcasses have low muscle development and have high subcutaneous fat deposition, which could impact farmer incomes [11]. In ewes, to optimize feed conversion rate and body weight gain (BWG) and increase meat dressing, the supply of energy-rich diets for short periods enhances animal performance, carcass traits, and meat quality [8]. The hypothesis for this study was that BWG, carcass yield, meat color, shear force, cathepsins activity, myoglobin content and protein content are improved parameters for young ewe's compared to cull ewes, but a strategy of re-feeding with high gran diets could increase productive parameters and meat quality of cull ewes. Therefore, our objective was to determine the influence of re-feeding with commercial concentrate diet (CD) and contrast group feeding only with alfalfa hay (AH) comparing cull ewes and yearling ewes on growth performance, carcass yield, and meat quality.

## 2 Materials and methods

### 2.1 Study site

The study was conducted at the experimental sheep facilities of the Facultad de Agronomía y Veterinaria of Universidad Autónoma de San Luis Potosí, Soledad de Graciano Sánchez, San Luis Potosí, México (Latitude 22°14′0.58″; Longitude 100°50′48.5″).

### 2.2 Animals and management

Animal procedures were reviewed and approved by the Committee for the Ethical Use of Animals in Experiments of the Universidad Autónoma de San Luis Potosi (MCA-2018-034). Forty Rambouillet ewes with a mean body weight (BW) (49.73 ± 4.41 to cull ewes and 39.19 ± 5.1 for yearling ewes) were assigned to one of two fattening treatments. Ewes were grouped by age: twenty yearling ewes (10 ± 0.23 months) and twenty cull ewes (60 ± 1.8 months). Cull ewes were going to be discarded due to their advanced age and they were separated from their offspring two months earlier. Ewes were fed with two types of diets: ground (2 mm) alfalfa hay (AH); and commercial concentrate diet (CD; Borrego Engorda; Vali Group®, México) containing sorghum grain (35 %), corn (35 %), soybean oil (3 %), soybean meal (12 %), vitamins and minerals (1 %) and fiber source (14 %). Treatments were: a) 10 cull ewes were fed only with AH, b) 10 yearling ewes were fed only with AH, c) 10 cull ewes were fed with CD, d) 10 yearling ewes were fed with CD. The chemical composition of the experimental diets is shown in Table 1. At 08:00 and 15:00 h, the ewes were fed with their respective diets. All ewes had free access to feed to ensure 100 g of orts per kg of the amount fed daily. At the beginning of the study, the ewes were identified, weighed, and randomly placed on individual roofed metabolic cages (1.5 m^2^) equipped with feeders, drinkers, and elevated slatted floors.

**Table 1:** Chemical composition (% dry matter (DM) basis) of the concentrate diet and alfalfa hay fed to the ewes during fattening.

Components (%)	Concentrate Diet	Alfalfa hay
Dry matter	90	88.5
Crude protein	14	14.0
Ether extract	2.0	1.30
Neutral detergent fiber	33.8	46.2
Acid detergen fiber	18.7	26.1
Ash	10	12.0

### 2.3 Feed analysis

Every week diet samples were polled and analyzed according to AOAC [12]; for dry matter (DM, 981.10), crude protein (CP, 967.03), neutral detergent fiber (NDF), and acid detergent fiber (ADF) according to Van Soest et al. [13].

### 2.4 Growth trial

The growth trial lasted 35 days and the ewes had an adaptation period (10 d) to diets, before they were all housed in the same pen. Ewes received grass hay and alfalfa hay and were weighed at the onset and at the end of the study to determine average daily gain. Feed intake was recorded daily, and the feed conversion rate was expressed as the ratio of feed intake to average daily gain.

### 2.5 Slaughter procedures and carcass measurements

Before slaughter, shrunk body weight (SBW) was recorded, and then feed and water were withdrawn for 24 h. Ewes were slaughtered humanely following the Mexican Official Norms (NOM-08-ZOO, NOM-09-ZOO, and NOM-033-ZOO) established to slaughter and process animals for meat production. Immediately after slaughtering, the measurements of weights from the carcass were recorded. The carcasses were stored at 4 °C for 24 h, then a sample from the *Longissimus lumborum* (LTL) were taken from each carcass using a dot square grid of 75 mm^2^; all samples were stored individually in a vacuum bag at −20 °C for subsequent analysis.

Carcass classification was evaluated by trained personnel according to the Mexican carcass classification (NMX–FF–106-SCFI-2006), using a scale from 1 to 4: 1) México Extra, excellent conformation no classification; 2) México 1, excellent conformation; 3) México 2, poor conformation; and 4) no classification. The area of the LTL between the penultimate and last ribs was measured with a tape measure in the longitudinal and transverse axes of the rib eye, both at the coccygeal region. The carcass length was determined, from the atlanto-occipital joint to the first coccygeal vertebra. Leg length was taken from the proximal femoral epiphysis to the level of the tarsometatarsal joint. Rump width and chest width were assessed as reported by Colomer-Rocher et al. [14]. Dorsal fat was measured with a vernier scale on the 6th rib and 12th rib. The components not included in the carcass (trachea, lungs, heart, liver, spleen; full and empty digestive tract, head, skin, and feet) were weighed individually [15] for carcass yield calculations. Carcass performance included a) hot carcass weight, which was calculated by sectioning and weighing the head, legs, and skin. They were eviscerated with a cut from the sternum to the symphysis pubis, and the trachea, lungs, heart, liver, and spleen were separated. All components were weighed. The rumen and intestines were weighed full and empty to obtain the difference in the weight of the gastrointestinal contents; b) cold carcass weight was obtained after twenty-four (24) hours post-filling with the carcass storage at 4 °C. This was expressed as the weight obtained after airing losses, c) carcass yield (hot and cold), was reported as the dressing percentage ([dressed carcass weight/live weight] × 100).

### 2.6 Meat quality analysis

Samples of LTL (150 g) taken at post-mortem days 1, 5, and 10, and were used to determine quality parameters. Meat color was measured through the L*, a*, and b* parameters using the using the Minolta chroma Meter CM-2500. L* represents the light to dark color, a* represents green to red, and b* represents the blue and yellow tones. The equipment was calibrated with a blank before each measurement with an aperture size of 8 mm, an observer of 10° and, an illuminant D65, while the blooming time was 20 min. For color measurement, a 3 cm^3^ area of the sample was taken so that the measurement at post-mortem days 1, 5, and 10 would always be in the same place and thus be more representative. The color was taken in triplicate. The color change (ΔΕ) was calculated at ten days post-mortem, using the following equation:

$$\Delta E=\sqrt{\left(\left(\left(Lo-L*\right)+\left(a0-a*\right)+\left(b0-b*\right)\right)\right)}$$

where: L0, a0, and b0 are the color parameters of the sample at day one, and L*, a*, and b* represent the color parameters at day 5 and 10 of sample maturation.

To analyze meat tenderness, the cathepsin B and cathepsin B + L activities and myoglobin concentration were quantified on five- and ten-days post-mortem following methods from Etherington and Wardale [16] and Trout [17], respectively. Three determinations were performed for each sample. Myofibrillar protein was determined according to Joandel-Monier [18].

### 2.7 Statistical analyses

Data were analyzed using a MIXED procedure [19] as a complete randomized design with a 2 × 2 factorial arrangement of treatments. The model included the fixed effects of age (yearling or cull), diet type (AH and CD), and their interaction on growth performance, carcass yield, and meat quality. Meat color, cathepsins, myoglobin, and protein were analyzed as repeated measurements. On these models the Auto regressive covariate structure gave the lowest BIC. The significance of differences between the values obtained on different sampling dates was determined with the Tukey test.

## 3 Results

### 3.1 Productive traits

Productive traits and carcass yield are shown in Table 2. Feed intake was higher (P ≤ 0.05) in ewes offered only concentrate. There was no significant (P = 0.08) change for the age effect cull ewes consuming more feed compared to yearling ewes. Daily weight gain was not affected (P = 0.08) by concentrated diets. Feed conversion (FC) had no differences. Carcass classification was similar between treatments.

**Table 2:** Feedlot performance of culls and yearling ewes finished with two types of diets.

Item	Treatments				SEM	*P*-value		
	Alfalfa hay		Concentrate diet					
	Culls	Yearlings	Culls	Yearlings		Age	Diet	Age × Diet
Initial weight, kg	49.3	37.2	50.1	41.1	6.08	0.01	0.32	0.23
Final weight, kg	53.2^a^	40.8^c^	55.9^a^	46.2^b^	2.20	0.01	0.21	0.15
Average daily gain, g/d	112^b^	102^b^	166^a^	145^a^	29.0	0.53	0.06	0.84
Dry matter intake, kg/d	1.32^b^	1.32^b^	1.82^a^	1.71^a^	0.27	0.52	0.01	0.58
Feed conversion	11.7	10.8	10.9	11.7	2.25	0.37	0.64	0.79

SEM = Standard Error of the Mean; ^a–c^ Means within a row with different superscripts differ (*P < 0.05*).

### 3.2 Carcass characteristics

Hot carcass weight was affected with diet and age (P = 0.02 and P = 0.04, respectively). Likewise, cold carcass weight and run-off were affected by diet (P ≤ 0.05), while carcass yield was affected by age (P = 0.01) and diet (P = 0.01) (Table 3). However, LTL area was higher for cull ewes respect yearling, thorax circumference, differed among treatments (P ≤ 0.05).

**Table 3:** Carcass characteristics of culls and yearling ewes finished with two types of diets.

Item	Treatments				SEM	*P*-value		
	Alfalfa hay		Concentrate diet					
	Culls	Yearlings	Culls	Yearlings		Age	Diet	Age × Diet
*Carcass Performance*								
Hot carcass weight, kg	21.3^ab^	17.8^b^	25.8^a^	21.7^ab^	1.19	0.04	0.02	0.85
Cold carcass weight, kg	20.6	17.6	22.2	20.6	1.50	0.36	0.05	0.26
Hot carcass yield, % Cold carcass yield, % Cooler shrinkage, kg	42.14 40.1 0.7^c^	46.36 45.40 0.2^c^	48.67 43.44 3.6^a^	49.44 45.7 1.1^b^	1.56 1.29 0.52	0.01 0.04 0.16	0.01 0.17 0.02	0.04 0.24 0.70
Carcass dressing, %	38.3^c^	42.1^bc^	43.7^b^	48.5^a^	1.32	0.01	0.01	0.56
Carcass length, cm	78.0	83.6	82.6	77.3	8.38	0.97	0.86	0.29
Area *Longissimus thoracis et lumborum,* mm^2^	67.9^a^	50.9^c^	69.9^a^	59.5^b^	10.99	0.23	0.63	0.76
Leg length, cm	44.3	45.3	43.3	42.6	2.08	0.95	0.25	0.58
Rump width, cm	19.0	20.6	19.0	19.0	1.15	0.36	0.38	0.36
Chest width, cm	27.3	30.6	29.6	28.0	1.52	0.69	0.93	0.25
Torax circumference, cm	83.6^b^	92.0^a^	85.0^b^	82.6^b^	10.4	0.67	0.58	0.04
Dorsal fat, mm	6.66^a^	2.87^c^	3.73^bc^	4.69^b^	1.74	0.3	0.67	0.1
Fat-tailed, kg	3.00	3.33	3.00	2.66	0.6	1	0.39	0.39
Carcass classification scale	3.66	3.33	3.00	2.90	0.57	0.8	0.46	0.8

SEM = Standard Error of the Mean; ^a–c^ Means within a row with different superscripts differ (*P < 0.05*).

### 3.3 Meat physicochemical characteristics

The pH at 45 min and 24 h, carcass length, leg length, leg width, thorax width, and thorax perimeter were not affected (P > 0.05) by treatments. Meat color parameters at 24 h had significant differences (P < 0.05; Table 4). On the contrary, a* increased (P < 0.05; Table 4) with animal age. On day five of maturation, L*, a*, b*, c* and h* were significant (P < 0.05; Table 4) affected by the diet, ΔE was affected by both age and diet (P < 0.05; Table 4). Cathepsins B, B + L, and Lowry protein content were similar among treatments. During day 10 of maturation, L*, b*, c*, and ΔE were different between treatments being higher for CD (P < 0 0.05; Table 4). Myoglobin concentration at 24 h, and days 5 and 10, showed a significant difference (P < 0 0.05; Table 4) between treatments, with effect by diet at 24 h decreasing with CD, and days 5 and 10 had an age effect.

**Table 4:** Meat physicochemical characteristics of culls and yearling ewes finished with two types of diets.

Item	Treatments				SEM	*P*-value		
	Alfalfa hay		Concentrate diet			Age	Diet	Age × Diet
	Culls	Yearlings	Culls	Yearlings				
pH *Longissimus thoracis et lumborum*								
45 min	6.26	6.48	6.37	6.49	0.23	0.24	0.64	0.72
24 h	6.19	6.32	6.52	6.32	0.33	0.16	0.19	0.79
Color 24 h								
L*	44.5^a^	38.1^b^	43.0^a^	41.7^a^	0.01	0.26	0.01	3.75
a*	10.7^b^	13.4^a^	10.5^b^	12.2^ab^	0.01	0.31	0.44	2.65
b*	17.5^a^	11.7^b^	13.4^b^	12.7^b^	0.01	0.02	0.01	2.55
C	17.5	17.8	17.1	17.5	0.61	0.68	0.83	1.12
H	52.8^a^	41.2^c^	51.8^a^	46.4^b^	0.01	0.05	0.01	2.03
Protein Lowry, mg/mL	7.48	7.02	6.27	8.54	0.22	0.82	0.08	0.77
Cathepsin B^¥^	3.82	2.92	4.66	2.37	0.05	0.83	3.34	0.84
Cathepsin B + L^¥^	3.63	2.34	3.8	1.86	0.03	0.81	0.63	0.75
Myoglobin, mg/mL	516	408	382	388	70.1	0.06	0.01	0.09
Color day 5								
L*	47.7^a^	38.9^c^	42.1^b^	42.4^b^	0.01	0.16	0.01	1.51
a*	7.59b	10.5a	8.85^ab^	9.60^a^	0.01	0.73	0.02	1.67
b*	15.6^a^	12.9^c^	13.8^b^	13.9^b^	0.01	0.10	0.01	0.63
C	17.4	16.7	16.5	16.9	0.62	0.28	0.05	1.35
H	63.9^a^	50.8^c^	57.6^b^	55.5^bc^	0.01	0.58	0.01	2.44
Protein Lowry, mg/mL	6.4	6.19	6.47	6.92	0.92	0.74	0.78	0.64
Cathepsin B^¥^	3.15	2.37	2.99	2.96	0.55	0.75	0.58	0.98
Cathepsin B + L^¥^	3.84	4.32	3.93	3.25	0.9	0.55	0.48	1.31
Myoglobin, mg/mL	272	465	351	379	38.8	0.01	0.89	0.01
Color day 10								
L*	47.7^a^	38.6^c^	46.4^a^	41.9^b^	0.01	0.19	0.01	2.79
a*	8.52^ab^	9.68^a^	7.12^c^	7.97^bc^	0.01	0.01	0.61	0.88
b*	16.0^a^	12.0^c^	14.6^ab^	13.0^bc^	0.01	0.75	0.02	1.86
C	18.1^a^	15.4^b^	16.3^b^	15.3^b^	0.01	0.03	0.06	1.05
H	61.9^ab^	51.2^c^	64.1^a^	58.1^b^	0.01	0.01	0.12	2.17
Protein Lowry, mg/mL	15.2	9.68	10.8	10.2	0.14	0.34	0.23	1.85
Cathepsin B^¥^	2.73	1.32	1.92	1.59	0.12	0.61	0.31	0.55
Cathepsin B + L^¥^	2.75	1.96	2.08	1.32	0.18	0.26	0.98	0.87
Myoglobin, mg/mL	264	368	292	278	41.4	0.04	0.16	0.01
ΔE day 5	4.34^bc^	3.69^b^	5.60^a^	4.65^ab^	0.06	0.01	0.72	1.24
ΔE day 10	3.71^b^	5.37^ab^	6.50^a^	5.45^ab^	0.57	0.01	0.01	1.04

SEM = Standard Error of the Mean; ^a–c^ Means within a row with different superscripts differ (*P < 0.05*); ^¥^ Expressed as specific activity in nmol of NMec (amino-methylcoumarin) released per min/mg protein.

## 4 Discussion

### 4.1 Productive traits

Feed intake is affected by weight, size, breed [20,21], and energy density. Pittroff and Kothmann [22] revised 11 models of feed intake where length, live weight, metabolic weight (PM0.75), and metabolic weight (PM0.73) were included. In our study, dry matter intake was modified (P ≤ 0.05) by the diet. In this regard, AH treatment generated ruminal distension [23], which was reflected on feed intake. These results differed from Jaborek et al. [24], who observed a higher feed intake in lambs fed solely with pelletized alfalfa compared to a diet based on whole shelled corn. These authors mentioned that energy density generates a compensatory intake that aid animals to cover physiological requirements, and this was also reported by Murphy et al. [25]. Both studies agree with our results. Mahgoub and Lu [26] mentioned that adult ewes could adapt better and have a higher intake with high forage diets due to enhanced rumen development. However, the feed intake with concentrate was higher than the alfalfa-based diet, while age did not influence this parameter.

Increasing concentrate levels in the ratio is proportional to the weight gain or growth rate in lambs [27]. McLeod and Baldwin [28] reported increased weight gains in lambs fed a diet containing 75 % concentrate than a diet containing 75 % forage. Similarly, in this study, ewes fed concentrate had higher weight gains. Age is a notorious factor where adult ewes had a higher numerically weight gain (131 g/adult vs. 123 g/young ewes). Cull ewes that are undernourished but are re-fed to meet or exceed their requirements can generate extraordinary weight gains [29]; Bohman [30] described this phenomenon as “compensatory weight” and that largely explain results from our study.

### 4.2 Carcass characteristics

The trend (P = 0.06) for diet effect could be because high concentrate diets decrease the acetate: propionate ratio [31], which may decrease losses due to caloric gain [32], making the use of energy for growth more efficient. Starch-sourced diets promote an elevated formation of ruminal propionate, which explains why gluconeogenic precursors can also induce feed and rumen efficiencies. Similarly, Lee-Rangel et al. [33] and Mendoza-Martinez et al. [34] reported a rise in total volatile fatty acids and propionic acid molar ratios. The present study focused on carcass characteristics and thus, rumen fluid was not obtained. However, if rumen fluid was taken results on volatile fatty acids would be similar to those reported in those previous studies.

Carcass yield is highly influenced by diet type [6], with differences between 40 and 50 % in yield, especially in animals fed high-grain diets. In hair sheep, carcass yield can fluctuate due to age, sex, physiological state, and feeding regime [2,4]. Extensive production systems generally have lower carcass yields due to gastrointestinal tract filling [3,6,8,35]. Although our animals were not in an extensive system, they were in a 100 % forage feeding regime and they indeed had a lower carcass yield (40.2 vs. 46.1, forage vs. concentrate).

Cacere et al. [36] found that live weight at slaughtering, empty weight, hot carcass yield, and cold carcass yield had a linear increase as dietary concentrate levels increased. Likewise, Ruiz-Ramos et al. [4] reported an increase in these parameters with animals fed on concentrate diets. Also, Fruet et al. [3] evaluated meat quality of cull ewes with a body condition of 3 raised under extensive systems or fed with ground grains-based diets and reported that there were no significant differences between treatments.

Bhatt et al. [37] reported that adding dietary fat in Malpura ewes led to increased carcass yields from 47.7 to 52.4 %. However, when evaluating the effect of level and time of fat supplementation in adult ewes, and hot carcass yield was not affected, and the average hot carcass yield (40 %) was lower than of the one obtained in our study. Though, Bhatt et al. [6] reported hot carcass yield values of 41 % and 48 % for animals in extensive and intensive systems, respectively, which agrees with the data from the present study.

The LTL area in our study was smaller than that reported in previous studies. Sebsibe et al. [38] mentioned that adult animals generally have a lower propensity of muscle to fat and bone. Breed can also influence the muscle development of the animal [39] and probably that factor explain the differences between previous studies and our data.

### 4.3 Meat physicochemical characteristics

Etherington and Wardale [16] mentioned that cathepsin B + L activity is greater in young animals than in adults. Those results contradict what was found in the present study, where the activity of cathepsins was higher in adult females than young females in cathepsins B and B + L at zero days. Hernandez et al. [40] reported that in pigs of 6 and 12 months the concentration of cathepsins in muscle was different. Likewise, Rosell and Toldra [41] observed differences in proteolytic enzyme activity between Iberian pigs and age at slaughter. Activity of cathepsin B and L in this study may be also influenced by feeding regime but mechanisms involved are still unknown.

Color is an essential parameter because it affects consumer's perception, and it is a quality reference because it is associated with freshness. Changes in myoglobin at 24 h and L*, b* and h* were affected by the diet; in this sense, color parameters are affected by myoglobin concentration, lipid oxidation, tissue structure, pH, and marbling [42]. Brightness is influenced positively by intramuscular fat and tissue water contents [43]. In this regard, a* values are generally affected by age at slaughtering [42] that only those observed at 10 days. Luciano et al. [44] mentioned that consumers relate red color with freshness, and changes in redness and yellowness over time have been described as a change from red to brown, which mirrors myoglobin concentration and the redox state in meat [42].

Meat from cull ewes had lower L*, a*, and b* values from day zero to day ten post-slaughter. Jaborek et al. [45] reported that LTL meat from mature ewes had higher L*, a*, and b* values than that of 12-month-old ewes, and regarding age, higher values were also reported in meat from 14- to 22-month-old sheep compared to meat from 8-month-old sheep [46]. Similar results were reported by Preziuso and Russo [47] and Bures and Barton [48]. Conversely, De Palo et al. [42], found higher values for 18-month-old donkeys and for 12-month-old donkeys, which is consistent with Funghi et al. [49], who obtained lower values of L* as the age at slaughtering increases in young bulls from 6 to 24 months. In lambs, Khliji et al. [50] reported that the ability of a* to predict consumer acceptance was around of 14.5 at 24 h which was higher compared to the present study. This same study also identified L* as an important predictor for lamb meat color preference, which is an outcome reflected by preliminary research assessing beef which concluded that L* was a potentially suitable candidate to be employed as a threshold value [51]. The disagreement between results from these and the present study may be due to differences in chroma precision.

Ewes that consumed concentrate had higher CIELAB values. The color of fat surface in lambs fed 100 % corn had lower L* values than lambs at 85 % of their theoretical requirement [45]. These results contrast with that reported by Karaca et al. [52], where higher L* and a* values in meat from lambs fed a concentrate ration in stall relative to lambs fed on pasture. Minchin et al. [53] reported that the brighter appearance of meat from cows fed with high-energy diets can be due to changes caused in reflectance values by increased fat deposition. Although, Priolo et al. [54] reported that the meat color was darker in pasture-fed lambs, even in conditions where fat deposition was high, when compared to concentrate-fed lambs.

Another important consideration in pasture-fed lambs is the meat redness or the *a*∗ value, which is associated with raised pigmentation that results from increased muscle activity [55]. It is important to note that meat lightness is inversely correlated with iron content, which increases with age [48]. Also, the CIELAB values are affected by myoglobin contents in muscle [56] and myoglobin concentration increases during the first two years of life [57]. Meat color from 6-month-old animals is less red due to low iron concentrations; however, meat from 18-month-old animals is rich in myoglobin, resulting in a darker product [45]. Color changes (ΔE) are those from lightness, bright red, and yellow fibrillary [58]. Although the differences in a* determine the differences in ΔE; also, the changes in L* and b* could contribute to the ΔE value. Those parameters that are more susceptible to changes during the oxidation process in meat are a* and c* [58]. In our study, changes in ΔE were observed on days 5 and 10, which agree with the qualification made by the National Bureau of Standards (NBS), and the ΔE change was observed in both days. Also, the Meat Color Measurement Guidelines of American Meat Science Association [59] mentioned that ΔE of less than 1.0 are not detectable unless the samples are side by side. These differences are constantly related to frozen meat [60], which may be due to the variations in the long-chain fatty acids content affecting the susceptibility of the meat to be oxidized during storage. In fact, color changes are also associated with the oxidation process and meat shelf life [58]. Thus, meat with a greater concentration of long-chain fatty acids increases the shelf life, reduces the meat's oxidation process, and positively relates to the value of ΔE and the sensorial tests [60]. Our data suggest that age and diet are related with changes in long-chain fatty acids contents, and ΔE is influenced by both age and diet. Further studies should analyze meat fatty acid profile to confirm our results.

## 5 Conclusion

Overall, the concentrate diet increased the average daily gain and dry matter intake regardless of the age. Nevertheless, yearling ewe's hag improved carcass yield even though cull ewes had a heavier hot carcass when fed with CD. Age increases the CIELAB values at day 0 of slaughter. At days 5 and 10, CIELAB values increased due to diet. The color changes increased with age at five days post-mortem, but at ten days, diet negatively affected it. In conclusion, feeding cull ewes with concentrate diets could improve body weight gain and carcass yield compared to alfalfa hay feeding.

## Ethics statement

The animal study was reviewed and approved by 10.13039/501100005324Universidad Autónoma de San Luis Potosi Ethics Committee (MCA-2018-034). Written informed consent was obtained from the owners for the participation of their animals in this study.

## Data availability statement

Data will be made available on request.

## Declaration of competing interest

We wish to confirm that there are no known conflicts of interest associated with this publication and there has been no significant financial support for this work that could have influenced its outcome.
